# Targeting Lung Cancer Stem Cells: Research and Clinical Impacts

**DOI:** 10.3389/fonc.2017.00080

**Published:** 2017-05-05

**Authors:** Norashikin Zakaria, Nazilah Abdul Satar, Noor Hanis Abu Halim, Siti Hawa Ngalim, Narazah Mohd Yusoff, Juntang Lin, Badrul Hisham Yahaya

**Affiliations:** ^1^Regenerative Medicine Cluster, Advanced Medical and Dental Institute (AMDI), Universiti Sains Malaysia, Kepala Batas, Penang, Malaysia; ^2^College of Life Science and Technology, Xinxiang Medical University (XXMU), Xinxiang, China; ^3^College of Biomedical Engineering, Xinxiang Medical University (XXMU), Xinxiang, China

**Keywords:** lung cancer, non-small cell lung cancer, cancer stem cells, neoplastic stem cells, surface markers, therapeutics

## Abstract

Lung cancer is the most common cancer worldwide, accounting for 1.8 million new cases and 1.6 million deaths in 2012. Non-small cell lung cancer (NSCLC), which is one of two types of lung cancer, accounts for 85–90% of all lung cancers. Despite advances in therapy, lung cancer still remains a leading cause of death. Cancer relapse and dissemination after treatment indicates the existence of a niche of cancer cells that are not fully eradicated by current therapies. These chemoresistant populations of cancer cells are called cancer stem cells (CSCs) because they possess the self-renewal and differentiation capabilities similar to those of normal stem cells. Targeting the niche of CSCs in combination with chemotherapy might provide a promising strategy to eradicate these cells. Thus, understanding the characteristics of CSCs has become a focus of studies of NSCLC therapies.

## Introduction

Lung cancer has remained as the leading cancer cases worldwide in term of the high incidence and mortality rate (1). Based on data published in GLOBOCAN, 2012, there are 1.8 million new cases of lung cancer (or 12.8% in total) and 1.6 million deaths (19.4%) in 2012 (1). In 2016, the number of new lung cancer diagnosed was increased to 14% and remained as the highest mortality rate accounting for about 1 in 4 cancer deaths (2). Based on pathological features, lung cancer has been classified into two major groups: small cell lung cancer (SCLC) that accounts for only 15% of all lung cancer cases and non-small cell lung cancer (NSCLC) that accounts for 85% of lung cancer cases. NSCLC is further classified into three subtypes: adenocarcinoma, squamous cell carcinoma, and large cell carcinoma (LCC). Due to very poor prognosis (only 15% of patients surviving 5 years after treatment), treatment for lung cancer remain challenging.

Researchers believe that there is a presence of small subpopulation of cells within the tumor cells that driven the aggressive behavior of cancer cells and chemotherapy resistance of the cancer cells. The subpopulation of cancer cells is known as cancer stem cells (CSCs) or cancer-initiating cells (CICs) (3). According to American Association for Cancer Research, CSC was defined as “a cell within a tumour that possesses the capacity to self-renew and to cause the heterogeneous lineages of cancer cells that comprise the tumour” (4). The theory of CSC suggests that this subpopulation of cells has the capacity to self-renew, initiate tumors, and undergo multipotent differentiation (3). Many of the concepts that arise in cancer research, such as self-renewal, heterogeneity, and relapse after treatment and resistance to conventional chemotherapies, can be explained by this theory.

The first solid evidence to support the CSC theory was the identification of relatively rare population (1:250,000 cells) of stem-like cells in acute myeloid leukemia (AML) (5). The cells were isolated based on the expression of surface protein markers and able to re-grown human AML when transplanted in immuno-compromised mice (5). Later, the analysis of stem-like cells from various AML subtypes has found that the cells were immature in terms of differentiation and closely related to the hematopoietic stem cells (HSCs) rather than mature, terminally differentiated blood cells (6). This breakthrough finding has fostered an intense effort to characterize and isolate CSCs in various solid tumor including breast, brain, prostate, colon, and pancreatic cancer (7–11) thus making the CSCs field at the rapidly evolving field that may play a pivotal role in changing how basic cancer researchers, clinical investigators, physicians, and cancer patients view cancer the CSCs study.

## Biology of CSCs

**Figure 1:**
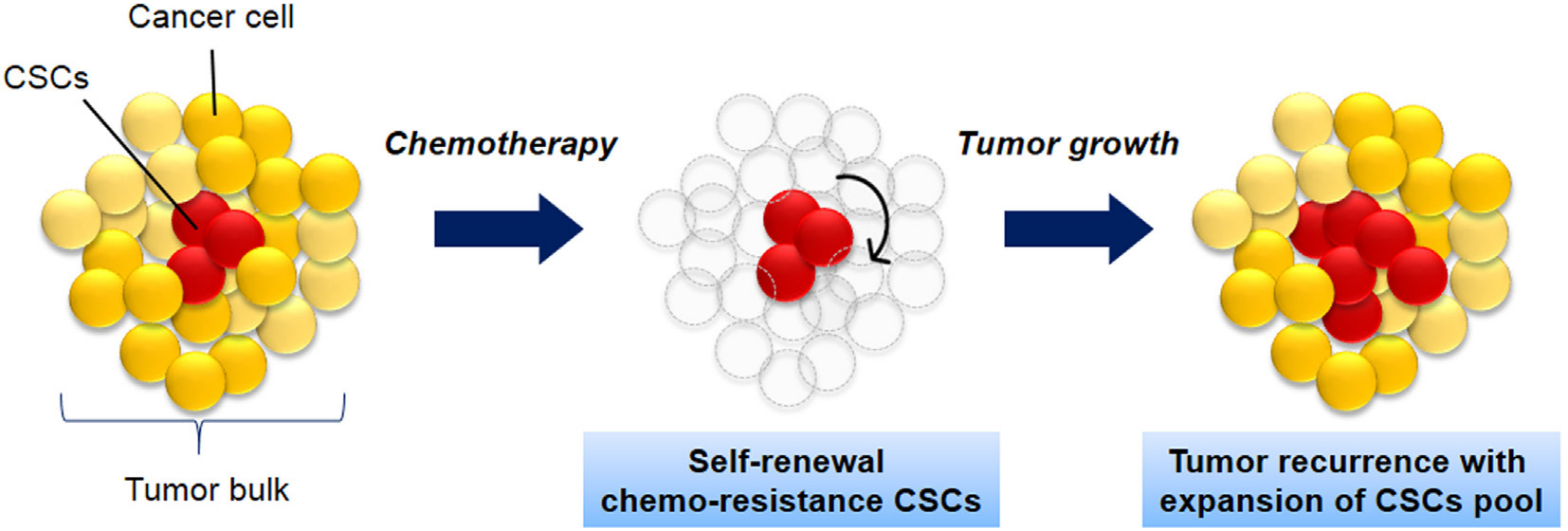
**The biology of cancer stem cells (CSCs)**. CSCs (red) self-renew and differentiate within tumors to form CSCs pool and non-tumorigenic cancer cells (yellow), which have limited proliferative potential. As the tumor grows, these cells can either undergo limited benign growth or form disseminated malignancies. These cells are resistance to chemotherapy and leads to cancer recurrence.

CSCs is defined as a small population of cells within tumor with share characteristics of normal stem cells. The cells have the capacity to initiate tumor formation, extensive proliferation, and resistant to chemotherapy (Figure 1). Apart from that, CSCs also share other similar characteristics like normal stem cells including self-renewal, expression of specific markers and genes, and utilization of common signaling pathways (3). The source of CSCs can be either from somatic stem cells or differentiated progenitor cells (3, 12, 13). Those cells initiate tumorigenesis by undergoing self-renewal and differentiation and thus resulting in tumor relapse, therapy resistance, and metastasis (3, 14).

CSCs are identified based on several approaches: (1) ability to self-renewal (sphere and colony forming assay), (2) ability to differentiate, and (3) ability to form secondary or tertiary tumors when transplanted into immunodeficient rodent (15). The ability of the CSCs to form tumor in immunodeficient mice was the commonest method to characterize the human CSCs. For instance, Dick et al. (16) had reported that the CD34^+^CD38^−^ cells derived from human leukemia cells, which constituted less that 1% cells in 10,000 leukemia cells had the ability to form leukemia in non-obese diabetic/severe combined immunodeficient (NOD/SCID) mice (16).

Similar to normal cells, tumors are composed of heterogeneous populations (17), which distinct in tumor subtypes. The heterogeneous tumor cells are different in terms of morphological and phenotypic profiles including proliferation and differentiation capacities. To illustrate, the progression of each cells within the tumor are different individually, in which there are some cells classified as cycling or non-cycling tumor cells, some may be in dormant state or reproductively dead (18). If the cells are in cycling mode, therefore, the cells may be at any stage of cell cycle which coincidentally may influence the cellular properties such as membrane properties (19, 20), antigen expression (21), sensitivity to immune killing (22), drug cytotoxicity (23), and ability to metastasize (24). Therefore, the characteristics of the tumor cells will be dependent on these properties. Back then, the heterogeneity of tumor cells may also eventually arise from genetic changes, environmental differences, and reversible changes in cells properties that resulted different in terms of morphology and biology of the tumor cells (25). Unfortunately, the diversity of tumor heterogeneity results in greater challenge in targeting specific CSCs. Thus, a number of specific markers are required in order to identify the CSCs population specifically lung CSCs (see Markers for Lung CSCs). Taken into consideration the tumor niches or microenvironment that control the stemness of the CSCs, the differences in the histological types and clinical presentations of lung cancer have contributed to the fact that the CSCs can produce morphologically and biologically different tumor within the same tissue.

Lung cancer has been considered as the most complex type of cancer due to the genotypic and histological varieties. This in turn, making the study of lung CSCs less explored as compared to other types of cancer (26). CSCs of lung cancer has been proposed to originate from several sources including from airway stem cells, bronchiole alveolar progenitor cells, basal/mucous secretary bronchial progenitor cells, or neuroendocrine progenitor cells. The different origin has resulting in the development of region-specific lung cancers (27). The study of CSCs has to be specific for respective subtypes of lung cancer and therefore making the CSCs study in lung cancer become more challenging.

The concept of CSCs in human lung cancer was initially coined 30 years ago (28). The study by Carney et al. (28) demonstrated that less than 1.5% of cells taken from patients with adenocarcinoma (SCLC) were able to form colonies when cultured *in vitro*. The selected colonies were able to generate the formation of tumor with similar features of the original tumor when transplanted into nude mice. Another study done by Kim et al. (29) has identified the bronchioalveolar stem cells from the mouse bronchioalveolar duct. They found out that the cells could initiate the development of lung adenocarcinoma (29). Recently, our group has identified the lung CSCs in NSCLC that was isolated from lung adenocarcinoma cells (A549 and H2170). Apart from showing characteristics similar to multipotent stem cells, the microarray analysis of the isolated lung CSCs also showed that the cells possess the biological characteristics associated with cancer and stem cells (30).

## Methods for Identification of Lung CSCs

CSCs have been identify and isolated using functional assay such as side population (SP) assay and based on the expression of CSCs surface marker.

### SP Assay

The SP assay method discriminate cells based on the differential potential of cells to efflux fluorescent Hoechst dye (a DNA-binding dye) *via* the ATP-binding cassette (ABC) transporters (31). The ABC transporter proteins are expressed within cell membrane, and they belong to the superfamily of membrane pumps that catalyze the adenosine triphosphate (ATP) and transport various endogenous compounds out of the cells (32). In human, it has been estimated between 500 and 1,200 genes encoded for drug transporter protein (33). Among them, the P-glycoprotein (ABCB1, MCR1), ABCG2 (breast cancer resistant protein, BRCP1), and ABCC1–5 (multidrug-resistant proteins, MRP1–5) are the drug transporter proteins involved in the establishment of SP phenotype (32). The SP assay was first described by Goodell et al. (34) for isolation of mouse bone marrow cells and shown to be enriched with HSCs (34). Moreover, the SP cells are overlapping with HSC phenotype CD177^+^Sca1^+^Lin^−^Thy1^−^ (35, 36). The SP cells have been described in various tumor types as being enriched in stem-like properties (37, 38). The SP assay was the first approach used for the identification of lung CSCs (39, 40). The SP cells isolated from established lung cancer cell lines are more tumorigenic and display increased invasive capability compared to the non-SP cells (39). Moreover, the cells are resistance toward multiple chemotherapeutic drugs and display high expression of ABCG2 and other ABC transporters (39). The SP cells also exhibit the self-renewal characteristics display by the ability to generate floating spheres and poses high proliferative potential (41).

### Surface Marker Expression

Another strategy used to identify lung CSCs is by expression of stem cell surface marker. Currently, there are relatively few lung CSC markers that have been validated. However, extensive studies have led to the identification of various CSCs that differ from other cells in the tumor. Most CSCs express multiple markers at the same time and using one marker to define CSC is not possible (Table 1). For example, in cell lines A549 and H446, CD133-positive and -negative populations contain the same amount of CSCs. Studying CSC markers may present new insight that will improve current lung cancer therapy and better patient prognosis.

**Table 1:** **List of markers used for identification of CSC in various tumor**.

Markers	Tumor	Reference
CD133 (prominin-1)	Lung, brain, and colon	(8, 42–44)
CD44 (membrane-bound glycoprotein)	Lung	(45)
Aldehyde dehydrogenases	Lung, leukemia, liver, pancreas, breast, and colon cancers	(46–49)
CD133^+^ESA^+^	Lung	(50)
CD90	Lung	(51)
CD87 (uPAR)	Lung	(52)
Side population	Lung	(39)
CD166^+^CD44^+^ and CD166^+^EpCAM^+^	Lung	(30)

#### CD133

CD133, also known as prominin-1, is one of the most commonly used markers for detecting CSCs. CD133 is a five-transmembrane glycoprotein that has been demonstrated to be highly expressed in various carcinomas of various origins including brain (8) and colon (42). The exact role of CD133 in lung cancer is still unclear. However the CD133^+^ cells are thought to be CICs on the basis of their ability to induce tumor development, invasion, and metastases. Previous study on NSCLC showed that CD133^+^ cells isolated from NSCLC tumor specimens have higher self-renewal capacity compared to CD133^−^ cells with 40-fold higher number of colonies/spheres formed. Self-renewal capacity of CD133^+^ and CD133^−^ cells were further confirmed by injecting the cells into NOD/SCID mice. The results had demonstrated that CD133^+^ cells possessed more tumourigenicity compared to CD133^−^ cells (44). Moreover, in tumor specimens of NSCLC patients, significant increase in CD133^+^ cells and capillary structure have been detected, suggesting the involvement of this cell population in tumor growth and tumor vasculogenesis (44). However, another study on NSCLC patients’ exhibit opposite findings where CD133 cells were found to acts as resistant phenotype, not as a prognostic marker for survival like previously reported (53). Salnikov and colleagues reported that even though CD133 was found in various lung cancer cell lines, including A549, H157, H226, Calu-1, H292, and H446, the CD133 was only exhibited CSC characteristics such as self-renewal, differentiation, proliferation, and tumorigenic capacity in H446 cell line. In another study, Eramo and colleagues were able to isolate small niche of CD133^+^ from SCLC and NSCLC and found that both the CD133^+/−^ populations have the ability to self-renew, but the CD133^−^ population were non-tumorigenic (43).

#### CD44

Another marker that has been proposed as CSCs marker is the CD44. CD44 was initially proposed as CSCs marker for colorectal cancer (54–56). CD44 is a membrane-bound glycoprotein that plays several important roles including cell migration, cell adhesion, and modulation of cell–matrix interaction (57). Besides, CD44 has been reported to be associated with many cancer-mediated signaling pathways that explain the involvement of this marker in cancer initiation and enhancement. For example, CD44 acts as a coreceptor with epidermal growth factor receptor and as an indirect activator of the cell proliferation pathway through the ligand presentation (58). In addition, CD44 has been reported to enhance cancer cells invasiveness and multidrug resistance due to its association with mitogen-activated protein kinase-phosphatidyl inositol 3 kinase signaling pathway. This association led to the overexpression of urokinase plasminogen activator/urakinase plasminogen activator receptor that results in enhancing invasiveness and multidrug resistance of cancer cells (52). Profiling of the expression of several markers including CD44 from 10 NSCLC cell lines by using flow cytometry found that CD44^+^ from certain cancer cell lines acts as a tumor initiator marker in lung cancer cells when tested in both *in vitro* and *in vivo* (45).

#### CD166

CD166 is another CSCs that have been described as CSCs marker for lung cancer. While CD166 has been studied extensively in other solid tumor (59–61), little is known about CD166 expression and its function in lung cancer. The role of CD166 as marker for lung CSCs has been demonstrated by Zhang et al. (62) in their comprehensive study on the potential of different surface marker candidates (CD166, CD133, CD44, and EpCAM) for the identification of CSCs in NSCLC (62). The isolated CD166 shows higher self-renewal potential and initiates formation of *in vivo* xenograft. Moreover, CD166 population shows higher *in vivo* tumor initiating capacity in comparison to CD133^+^, CD44^+^, and EpCAM^+^ cells isolated from the same cells. Based on this evidence, CD166 marker is considered as the most robust CSCs marker for identification of lung cancer.

#### Aldehyde Dehydrogenase (ALDH)

Another marker for CSCs that recently caught the attention of researchers is *ALDHs*. *ALDH* superfamily is composed of 19 known functional genes that can be classified into 11 families and 4 subfamilies (63). *ALDH* superfamily is NADP^+^-dependent enzymes that play crucial role oxidation of aldehydes into carboxylic acid. In retinoic signaling, *ALDHs* are required to produce the active form of retinoic acid by oxidation of all-trans-retinal and 9-cis-retinal (64, 65). There are several isoforms of ALDH that play important role in retinoic acid signaling including *ALDH1A1, ALDH1A2, ALDH1A3*, and *ALDH8A1* (66). Several *ALDH* isoforms has been reported to involve in stem cell and also CSCs populations including *ALDH1A1, ALDH1A2, ALDH1A3, ALDH1L1*, and *ALDH1L2* (63). *ALDH* was found to be highly expressed in various cancers such as leukemia, liver, pancreas, breast, and colon cancers (46, 47, 67). Recently, *ALDH* have been recognized as common markers not only for normal but also for CSCs (63, 68, 69). Profiling of *ALDH* expression in 12 different human lung cancer cell lines by using flow cytometry-based assay exhibited high expression of the cytosolic form of *ALDH1A1* and *ALDH3A1* (70). The involvement of *ALDH* in CSCs was further clarified by Ucar and colleagues (49). In their study, they compare the proliferative potential between two subpopulations of CSCs: a population with bright *ALDH* activity and population with dim *ALDH* activity in H522, lung cancer cell line. The obtained results show that the cells’ population with bright *ALDH* activity has higher and long-term proliferative capability compared to cells population with dim *ALDH* activity (49). Consistent with this result, Jiang et al. (48) found that ALDH^+^ cells, isolated from human NCSLC cell lines had important properties of CSCs in both *in vitro* and *in vivo* studies (48). Further clarification of the specificity of *ALDH* in lung cancer development is needed, as *ALDH* expression in normal pneumocytes increases due to exposure to cigarette smoking (70). Both these *ALDH* isozymes have been reported to play crucial role in stem cells (63).

#### Other Markers

CSCs that were known to have a capability in self-renewal, differentiation, and tumor initiation might express multiple markers instead of only single marker. For example, in a study on lung cancer cell lines A549 and H446, it was proved that CD133 alone could not be used to isolate the CSCs since both CD133^+^ and CD133^−^ cell populations contained similar number of cells that contains CSCs characteristic (71). In another study on lung cancer cells, CSCs have been isolated by using combination CD133 with other markers such as ABCG2, chemokine receptor 4, and epithelial-specific antigen (ESA) (50). Population of CD133^+^ESA^+^ population was higher in NSCLC compared with normal lung tissue. Comparison between positive and negative populations of CD133 found that CD133^+^ population exhibit higher tumorigenic capacity when injected in SCID. Recently, our group had found that CD166^+^CD44^+^ and CD166^+^EpCAM^+^ cells isolated from A549 and H2170 lung cancer cells showed the stemness characteristics including the sphere and colony-forming capabilities, expression of stem cells transcription factors (Sox2 and Oct 3/4), and tumorigenic capacity when transplanted in nude mice (30, 72, 73). Thus, it is clearly shown that cancer cells contain a heterogeneous population of CSCs that expresses various cell surface markers and the use of more than one marker for isolating CSCs might increase the stringency of the CSCs population. However, it is still not isolating the whole population of CSCs in any cancer cell populations. Therefore, more research should be done in order to explore more markers that can be used to identify lung CSCs.

## Therapeutic Strategies of Lung CSCs

CSCs are believed to sustain the progenitor of cancer cells and regulate the progression of cancer development in human. Therefore, targeting CSCs may provide a strategic way in therapeutic of cancer. Signaling pathway such as Hedhgog (Hh) is function in development and regeneration or repairing of stem cells (74). However, due to mutation, the aberrant of Hh signaling pathway activated the oncogenic pathway and eventually lead to tumorigenesis including initiate the CSCs progression that is responsible in cancer relapse. Therefore, inhibit the Hh pathway would be a great strategic by aiming on (1) inhibition of ligand processing, (2) disruption of receptor ligand, and (3) inhibition of transcription factor, the glioma-associated oncogene transcription factors 1–3 (*GLI1, GLI2*, and *GLI3*) (74). In addition, Hh pathway is also associated with chemoresistance (75, 76), which is also a characteristic that defines the properties of CSCs and causes major failure in chemotherapy (77).

Understanding the biological properties of lung CSCs is a fundamental toward improvement of the therapeutic effectiveness in cancer therapy. M2 isoform of glycolytic enzyme pyruvate kinase (PKM2) is well known in regulating the tumorigenic of cancer and associated with therapeutic resistance (78) that eventually contributes to poor prognosis of cancer. A recent study has revealed that PKM2 expression was associated with the biological properties of lung CSCs in which when the function of PKM2 was silent in CD44^+^ cells, the capability of CSCs to form spheroid and colony were reduced, and the sensitivity toward cisplatin was increased (79). On the other hand, an activation of D_2_ dopamine (DA), a receptor in CD133^+^ adenocarcinoma NSCLC were significantly inhibited the proliferation, colonies formation, and invasiveness of this tumor cells (80). In a different study, the lysine demethylase 1 (LSD1) was discovered to play a role in maintaining SCLC stemness, thus could be a potential therapeutic candidate in targeting lung CSCs. Interestingly, a potent inhibitors for LSD1 known as GSK2879552 was discovered, which was found to be sensitive specifically to only SCLC and AML as compared to the other 165 cell lines that were tested (81). Therefore, it is essential to discover the biological properties of lung CSCs as a promising in selective targeted therapy.

Cellular surface markers are one of the common method uses in discriminating CSCs population. Markers such as CD166^+^/EpCAM^+^ and CD166^+^/CD44^+^ has been correlated to CSCs since it possesses the characteristics of CSCs including the ability to self-renewal, differentiated into adipogenic and osteogenic, and expressed transcriptomic profile of multipotent cells (30) that therefore could be a potential in targeting the CSCs population. Another strengthen markers that is CD44^High^CD90^+^ which eventually represent a CSCs population in SCLC and LCC as it capable on forming a spheroid that is recognized as tumor model for *in vitro* study (82). Furthermore, the clinical study showed that dual expression of stem cells markers, the CD133^+^ABCG2^+^ has showed early relapse in stage 1 NSCLC as compare to non-CD133^+^ABCG2^+^ (83), which will be useful target in cancer chemotherapeutic. Perhaps such marker as CD133 (44), CD44 (45), CD24 (84, 85), and CD166 (62) are known to detect lung CSCs but are also used in other study to detect CSCs in head and neck region (86, 87), gastric (88), colorectal cancer (89–92), breast (93), and pancreatic (94). Nevertheless, functional assay including SP and Aldelflour assay also has been used as a strategy to distinct between CSCs and non-CSCs. Based on Hoechst 33342 dye efflux assay, the cells will be isolated as it develop the ability of stem cells to efficiently efflux the dye and consequently represent a Hoechst 33342 dye negative and will be characterize as SP (41). Likewise, Adelflour assay was used to isolate cells that possess high *ALDH* enzymatic activity which in the same way mimic the capacity of stem cells (95, 96) that encode for resistance potential.

Natural product has widely use in cancer treatment as it contain low or fewer side effect but exhibit strong antitumor activity. Curcumin, a yellow-pigmented polyphenol, derived from *curcuma longa* (commonly known as turmeric) has been used in traditional Indian Ayurvedic as anti-inflammatory agent. Finally, scientist has showed that curcumin has chemopreventive and therapeutic properties against many tumors and enable it to suppress cell proliferation and inflammation, induce apoptosis, suppress many cancer signaling and pathway, inhibit cells metastasis and angiogenesis, and sensitize tumor cells to cancer therapies (97, 98). Curcumin has effectively sensitizes NSCLC CSCs and enhances cisplatin effectiveness thus induce cells apoptosis and suppression of cell migration of CD166^+^/EpCAM^+^ population (99), which subsequently reverse cisplatin resistant (100) in CSCs. Another recent study also shown that curcumin effectively inhibit the lung CSCs traits as evidence of shrinking the tumoursphere formation and also decreasing the expression of lung CSC marker as well as suppressing cells proliferation and induce cells apoptosis through a specific signaling known as Wnt/β-catenin and sonic Hedgehog pathways (101). Perhaps, not only lung CSCs but also curcumin has shown to efficiently inhibit several others types of cancer including colon cancer stem-like cells (102), breast CSCs (103), SP of C6 glioma cell (104), and CSC of colorectal liver metastasis (105).

The tumor microenvironment offer a niche that can sustain the growth (106) of CSCs as well as a protective role by sheltering the CSCs from any genotoxic insults, which results in therapy resistant (107). The niche possesses a complex structure (107) composed of diverse of stroma cells including mesenchymal and immune cells, vascular network, soluble factor extracellular matrix components, and also homeostatic processes (108), which is important in order to retain the abilities of CSCs to self-renewal and give rise to progenitor cells through differentiation process (109). Thus, interrupting CSCs niche would be necessary and could be a promising strategy for eradicating CSCs. Current study on targeting CSCs niche found that integrin, which is the primary receptors that involved in cell–matrix adhesion have a profound impact on the ability of CSCs to survive in specific locations. Recent findings demonstrated that the integrins, which play important role in CSCs biology, are required for cancer progression and drug resistance. Previous study on *K-ras* mouse model of NSCLC demonstrated that CSCs expressing integrin β4 are enriched after treatment with cisplatin (110). In consistent with this study, study on breast cancer showed that after treatment of cancer cell with taxol, the integrin α6^+^ CSC population is upregulated (111). Also, study of β1 integrins on head and neck cancer cell also support that integrins can act as potential cancer targets (112). However, specific integrins that is responsible to promote stemness, drug resistance, and metastasis of CSCs are yet to be determined (113). Recently, there are a few integrin α2-binding agents that have been invented to reduce the proliferation capability of CSCs (114). Therefore, it is clear that identifying specific integrins that is responsible to maintain survival of CSCs might be a promising target for cancer therapy.

Current technique of targeting CSCs is using miRNA therapy. microRNAs or miRNA are short nucleotide (~18–25 nt) of non-coding RNA that is responsible in regulation of gene expression by binding to 3′-untranslated regions or open reading frame of target genes (115). Formerly, the important characteristics that determine the CSCs are self-renewal, and study has shown that miRNA regulates this important feature. Miao et al. (116) discover that downregulation of *miR-27* enhanced the properties of stem-like SCLC, whereby downregulation of *miR-27* dedifferentiate non-tumorigenic cells into highly tumorigenic cells and was consistently downregulated in spheroid cells even at passage 4 (116). In addition, *miR-27* was also reported to be downregulated in A549 cell that derived from NSCLC type (117). There were many more miRNA that act as tumor suppressor such as miR-145 (118) and miRNA-34a (119) demonstrating the inhibition proliferation of lung cancer-initiating cells and epithelial–mesenchymal transition (118), the key step of metastasis of cells.

## Challenges in Lung CSCs

Overcoming the CSCs could be one of the strategic ways in increasing the therapeutic of cancer diseases as it is associated with poor prognosis. However, the fact that CSCs should be eliminated is not as simple as the theory as it faces many challenges and limitations. There are several things need to be aware and considered in order to improve the method in targeting the CSCs populations. The heterogeneity in CSCs, which results in diverse phenotype, has become a great challenge in identifying the prominent CSCs subpopulation especially in lung. Moreover, lung structure consist of complex structure comprise of a large variety of morphologies that differently function and responsible in facilitating gas exchange, balancing fluid in the lung, detoxifying and clearing foreign agents, and the activation of inflammatory due to injury (120, 121). Therefore, to track the CSCs, a list of markers has been widely select but is not always reliable because certain marker may not be specific in targeting the CSC. Study by Meng et al. (71) revealed that single marker of CD133^+^ showed no differences with CD133^−^ as both of these markers display similar CSCs features such as the ability of self-renewal, colony formation, differentiation, and invasion (71). Perhaps, during the selection of CSCs markers, there could be a contradiction in declaration of certain markers used, for example, Roudi et al. (122) mentioned CD44 and CD24 is not a potential CSCs used in A549 cell derived from NSCLC because both CD44^+^/CD24^+^ and CD44^+^CD24^low^ are capable of forming the holoclone, meraclone, and paraclone colonies and develop spheroid formation (122). Furthermore, cellular markers are difficult to stand alone and have to be used in combination of other markers in order to improve the selection of CSCs. However, targeting CSCs in lung would be challenge due to heterogeneity (123) of the cells and various genomic pathways involved. Therefore, many studies are focusing on using combination of cellular markers as it increase the specificity of targeted population.

CSCs are known to be resistant to chemotherapy and therefore are accountable for cancer relapse. This would be the major challenge in targeting CSCs as it usually causes failure in chemotherapy. CSCs usually display high expression of multidrug-resistant gene whose role involved in drug-efflux pump and is regulated by ABC transporter family such as *ABCB1* and *ABCB2* (77). Liu described CD133^+^ lung cancer cells exhibited drug resistance when treated with cisplatin with both *ABCG2* and *ABCB1* was upregulated and therefore increased the cross resistant to doxorubicin and paclitaxel that usually used for second-line agent (124). In addition, the enrichment of CSCs population were also reported in recent study, whereby the subpopulation of A549 and H2170 with markers CD166^+^/EpCAM^+^ showed higher expression upon cisplatin treatment as comparing to basal expression (99). In another study of NSCLC, the CD166^+^ cells with strong CSCs characteristics also were resistant when exposed to cisplatin drug (125). Furthermore, CSCs can adopt in quiescent state where it resist any cytotoxic insult (126) due to high expression of *Bcl-2/Bcl-XL* that function in mitochondrial ATP/ADP exchange as to prevent apoptosis which probably contributes to resistant of CSCs upon chemotherapy (127). Thus, in future, it is important to break the CSCs resistance properties as this controlled countless cytotoxic agents from entering the plasma membrane, which afterward cause relapse in cancer.

Current research is struggling in finding a selective ways to inhibit the CSCs and its characteristics. Though certain things need to be considered as CSCs share a similar characteristics to stem cells, whereby targeting the CSCs could also give effect to normal stem cells and toxic to health tissue. Therefore, a selective method have to be done to specifically aiming on CSCs without affect any others normal cells.

## Conclusion

As the second most common cancer in men and third most common cancer prevalence in Malaysians diagnosed each year, lung cancer is most complicated cancer diseases to treat due to chemodrug resistant and relapse. Therefore, the fundamental understanding on the basic science studies relating to CSCs development and maintenance are gaining momentum recently due to the fact that it has been known to be responsible to lead cancer progression, tumourigenicity, and therapeutic resistance. Thus, CSCs has become a novel and important clinical targets in cancer therapy in modern treatment. However, considering the fact that each type of tumor involves different epithelial/progenitor or stem cell types that controlled by various molecular pathways, variation in expression of the markers is even existent in the lung cancer subtypes making them all more difficult to be identified and targeted. Therefore, this review had discussed the importance and prospect of lung CSCs that would beneficial for future therapeutic of lung cancer.

## Author Contributions

All authors have contributed in writing and editing the manuscript text.

## Conflict of Interest Statement

The authors report no conflicts of interest. The authors alone are responsible for the content and writing of the article.
